# Warm Autoimmune Hemolytic Anemia Secondary to Babesia Microti Infection: A Case Report

**DOI:** 10.7759/cureus.50294

**Published:** 2023-12-10

**Authors:** Pavel Bleik, Vivian Matubia

**Affiliations:** 1 Internal Medicine, Mary Imogene Bassett Hospital, Cooperstown, USA

**Keywords:** tick-borne infection, babesia, warm autoimmune, hemolytic anemia, hematology

## Abstract

*Babesia microti* is a parasite endemic to the northeastern and midwestern regions of the United States of America and a leading cause of babesiosis. Babesiosis has a non-specific presentation, which can delay diagnosis, leading to increased morbidity and mortality. As the number of reported babesiosis cases increases, there is a need to create more awareness of some atypical presentations that allow for early recognition and treatment. This case report identifies a 75-year-old male with intact spleen who presented with warm autoimmune hemolytic anemia secondary to *B. microti* and had complete recovery within less than a month after treatment was initiated. We also briefly report on the known and suspected pathophysiology and treatment courses.

## Introduction

Autoimmune hemolytic anemia (AIHA) occurs as a result of antibodies formed against red blood cells (RBC), leading to premature destruction. Warm autoimmune hemolytic anemia (wAIHA) is the most common type of AIHA, 60%-70% of cases, with antibodies forming when body temperature is 37°C or higher [1]. wAIHA can arise spontaneously (idiopathic wAIHA) or in the setting of different medications/conditions that could make a person prone to autoantibody production (secondary wAIHA). Though rare, secondary wAIHA has been noted in cases of babesiosis.

Babesiosis is a tick-borne zoonotic disease prevalent primarily in the Northeast and Midwest regions of the United States of America [2,3]. Since 2011, the CDC has made babesiosis a reportable disease, allowing for a better understanding of epidemiology. It is noted that between 2011 and 2019, disease incidences increased by 25% in the endemic US regions [4]. Most infections are asymptomatic [5,6]. However, the nonspecific symptomatology of babesia can make diagnosis difficult, especially in regions where there is a lower incidence. Babesiosis may have a non-specific presentation, which can delay diagnosis, leading to increased morbidity and mortality. Babesia is more likely to be associated with hemolysis compared to other tick-borne illnesses. However, it is unusual for babesia-related hemolysis to be due to AIHA, particularly in immunocompetent patients with intact spleens, as reported in the below case.

## Case presentation

A 75-year-old dependent male living in his own house with a past medical history of atrial fibrillation, hyperlipidemia, coronary artery disease, benign prostatic hyperplasia, essential hypertension, and well-controlled type 2 diabetes mellitus without insulin use came to the emergency department (ED) with two weeks of progressive fatigue, lightheadedness, generalized joint pain, stomach discomfort, and an episode of chills. Joint pain was observed in the shoulder and hips, with no small joint involvement noted. The patient also reported associated difficulty urinating and left flank pain without hematuria.

Vitals were stable with the patient in ambient room air. The exam was notable for jaundice, conjunctival pallor, and mild left flank tenderness, with the remainder of the exam unremarkable.

Blood work was most notable for a mild decrease in sodium, elevated creatinine from the baseline of 1.1 mg/dL, and elevated bilirubin (Table 1) in the setting of normal liver function.

**Table 1:** Patient electrolyte panel and liver function tests at the time of presentation, with units and reference ranges provided. Data marked (H) means higher than the reference value and (L) means lower than the reference value. Mildly decreased sodium levels were noted. Elevated creatinine and decreased GFR are consistent with impaired renal function. Elevated indirect bilirubin despite liver enzymes and albumin within normal limits, which is consistent with hemolysis. ALB: Albumin, ALT: Alanine transferase, AST: Aspartate transferase, BUN: Blood urea nitrogen, CO2: Bicarbonate, GFR: Glomerular filtration rate

Electrolyte Panel		
	Reference Range & Units	Patient values
Sodium	136-145 mmol/L	132 (L)
Potassium	3.5-5.1 mmol/L	4.2
Chloride	98-110 mmol/L	102
Glucose, Random	70-139 mg/dL	238 (H)
Glycohemoglobin (A1c)	4.0-5.6 %	5.1
CO2	21-31 mmol/L	20 (L)
Anion Gap	6-14 mmol/L	10
BUN	7-25 mg/dL	28 (H)
Creatinine	0.7-1.3 mg/dL	1.4 (H)
GFR	>=60 mL/min/1.73m2	53 (L)
BUN/Creatinine Ratio	10-20	20
Calcium	8.6-10.3 mg/dL	8.7
Alkaline Phosphatase	34-104 U/L	70
Albumin	3.5-5.7 g/dL	4.0
Total Protein	6.0-8.3 g/dL	7.5
AST	13-39 U/L	34
ALT	7-72 U/L	17
Total Bilirubin	0.3-1.0 mg/dL	2.6 (H)
Bilirubin, Direct	0.0-0.2 mg/dL	0.5 (H)
Bilirubin, Indirect	0.0-0.9 mg/dL	2.1 (H)
Lipase	11-82 U/L	29

Complete blood count with noted acute anemia and mild thrombocytopenia, though leukocytes remained within normal limits (Table 2).

**Table 2:** Patient completes a blood count with a cell differential at the time of presentation, with units and reference ranges provided. Data marked (H) means higher than the reference value and (L) means lower than the reference value. The patient has low RBC and hemoglobin levels with increased reticulocytes, consistent with RBC loss, and an increased marrow response. The patient has low platelets as well. Leukocytes are within normal limits. MCH: Mean corpuscular hemoglobin, MCHC: Mean corpuscular hemoglobin concentration, MCV: Mean corpuscular volume, RBC: Red blood cell count, RDW: Red cell distribution width.

Complete Blood Count		
	Reference Range & Units	Patient values
White Blood Cell Count	3.7-10.6 x10E3 cells/uL	4.9
RBC	3.70-5.90 x10E6 cells/uL	2.66 (L)
Hemoglobin	11.5-18.0 g/dL	8.5 (L)
Hematocrit	35.0-50.0 %	25.4 (L)
MCV	81.0-99.0 fL	95.5
MCH	27.0-33.5 pg	32.0
MCHC	31.5-35.5 g/dL	33.5
RDW	37.3-49.0 fL	53.8 (H)
Platelet Count	140-425 x10E3 cells/uL	120 (L)
# Reticulocyte	0.04-0.12 x10E6 cells/uL	0.22 (H)
% Reticulocyte	0.6-2.3%	8.5 (H)
Immature Reticulocyte Fraction	2.3-15.9%	29.3 (H)
Neutrophils Relative	40.0-70.0%	63.7
Neutrophils Absolute	1,500-7,400 cells/uL	3,100
Lymphocytes Relative	12.0-50.0%	20.0
Lymphocytes Absolute	950-3,500 cells/uL	970
Monocytes	2.0-14.0%	13.0
Monocytes Absolute	150-940 cells/uL	630
Eosinophils Relative	0.0-6.0%	1.9
Eosinophils Absolute	0-550 cells/uL	90
Basophils Relative	0.0-3.0%	0.6
Basophils Absolute	0-175 cells/uL	30
Immature Granulocyte Relative	0.0-3.0%	0.8
Immature Granulocyte Absolute	0-150 cells/uL	40

The anemia work-up done for completion was remarkable for elevated ferritin with iron, folate, and B12, all within normal limits (Table 3). The hemolytic work-up was completed with an elevated reticulocyte count, elevated LDH, low haptoglobin, and elevated indirect bilirubin consistent with hemolysis (Table 3).

**Table 3:** Patient anemia and hemolysis work-up at time of presentation, with units and reference ranges provided. Data marked (H) means higher than the reference value and (L) means lower than the reference value. Low haptoglobin and elevated reticulocyte count in the setting of elevated LDH are consistent with hemolysis. Iron levels are within normal limits with elevated ferritin, which could be due to an acute inflammatory process. The RPI upper limit of normal indicates increased marrow activity consistent with red blood cell loss. LDH: Lactate dehydrogenase, RPI: Reticulocyte production index, TIBC: Total iron binding capacity

Anemia and Hemolysis Panel		
	Reference Range & Units	Patient values
Iron	50-212 ug/dL	66
Ferritin	16-243 ng/mL	619 (H)
Iron Saturation	20-50%	22
Transferrin	203-362 mg/dL	216
TIBC	250-450 ug/dL	302
Folate	5.9-24.8 ng/mL	22.0
Vitamin B12 Assay, S	180-914 pg/mL	242
# Reticulocyte	0.04-0.12 x10E6 cells/uL	0.22 (H)
% Reticulocyte	0.6-2.3%	8.5 (H)
RPI	%	2.4
Reticulocyte Hemoglobin Equivalent	30.8-36.6 pg	34.5
Immature Reticulocyte Fraction	2.3-15.9%	29.3 (H)
Haptoglobin, S	30-200 mg/dL	<14 (L)
LDH	140-271 U/L	599 (H)
D-Dimer, Quant	215-499 ng/mL FEU	482
Erythropoietin	2.6-18.5 mIU/mL	57.3 (H)

**Figure 1:**
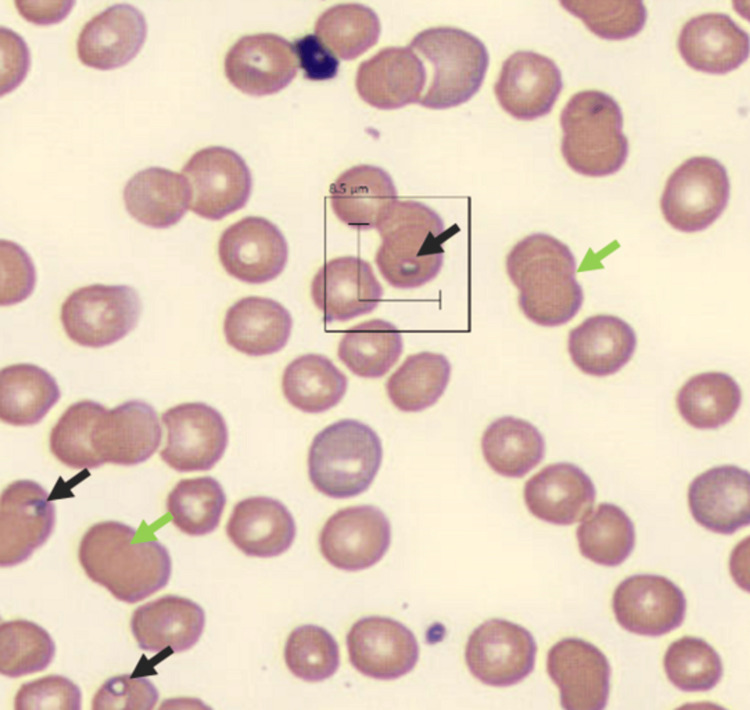
Image from the patient’s blood smear showing intracellular parasitic inclusions, merozoites (black arrows), and spherocytes (green arrows).

The type and screen were direct Coombs tests positive for IgG, only leading to increased suspicion of wAIHA. The evaluation of the blood smear showed intraerythrocytic parasitic inclusions and spherocytes (Figure 1).

The stool occult guaiac test was positive for occult blood, though no melena was noted. Urinalysis was positive for a moderate amount of blood, but there were only 3-5 RBC, which was possibly due to hemoglobinuria related to hemolysis.

A tick-borne PCR panel and Lyme titer were obtained due to the patient spending time outdoors in an endemic area. PCR positive for *B. microti* and negative for co-infection with *Anaplasma spp.*, Lyme disease, or *Ehrlichia spp*. Babesia parasitemia of 0.3% was reported from a sample sent to an external hospital laboratory.

**Figure 2:**
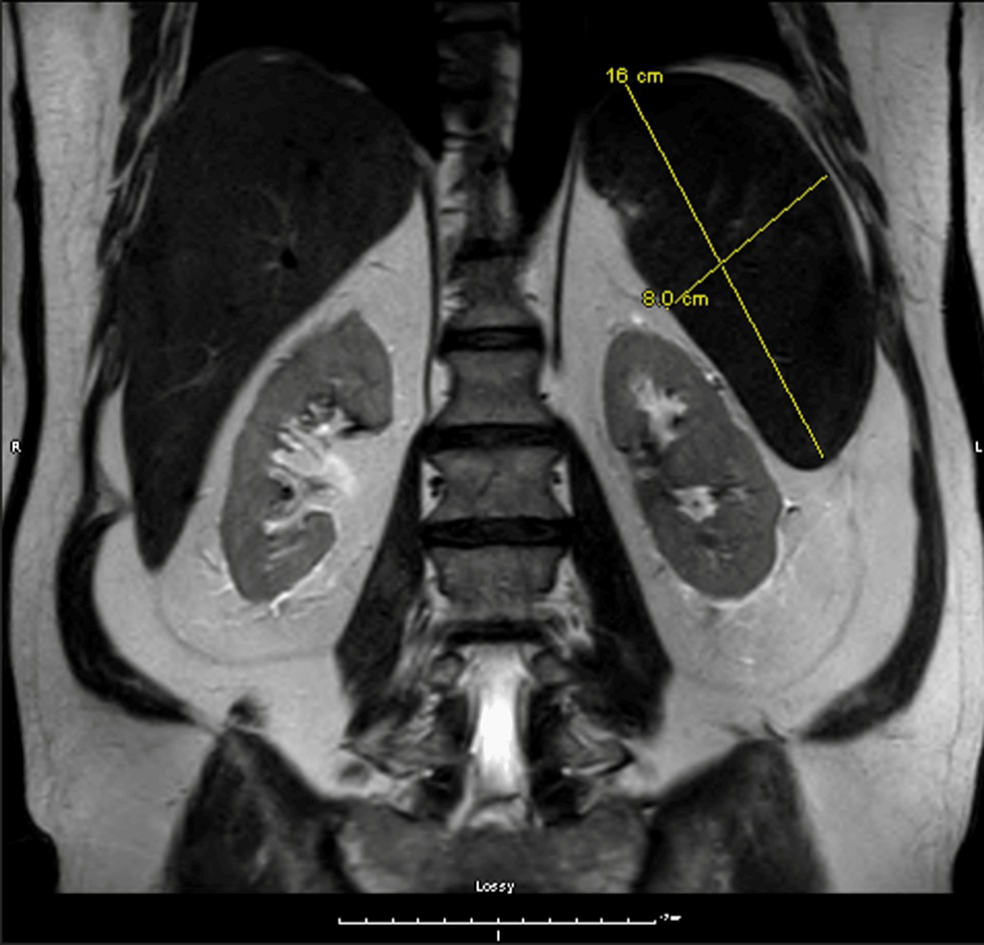
A T2-weighted TSC-corrected MRI image indicates splenomegaly and no identifiable renal mass.

The electrocardiogram showed a normal sinus rhythm without any noted abnormalities. The chest X-ray was only significant for basilar atelectasis. Further evaluation for lymphadenopathy was done to rule out occult malignancies. The CT chest with contrast was unremarkable. On a CT scan of the abdomen, the patient was found to have splenomegaly and an indeterminate renal mass. MRI was done to further evaluate renal mass, but remonstrated splenomegaly and reported mild hepatomegaly with no renal mass noted (Figure 2).

Based on blood smears, splenomegaly, Coombs positivity for IgG-hemolytic anemia, and tick-borne panel positivity for B. microti, our final diagnosis was wAIHA due to babesiosis.

The patient was started on atovaquone 750 mg daily and azithromycin 500 mg once, followed by 250 mg daily for a total of 10 days. Hematology was contacted, and the patient was started on Prednisone 90 mg, but his Hb continued to drop and reached 7.9; he got one unit of blood, and his Hb went up to 9.3.

**Figure 3:**
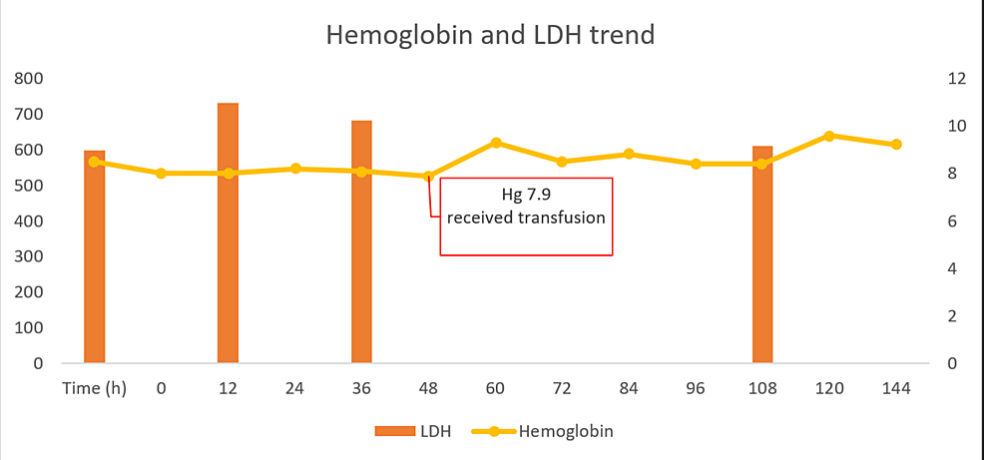
Graph showing hemoglobin trend (line) over time, with the right column indicating values in milligrams per deciliter. LDH levels (bars) are also shown with the units in the left column in units per liter. LDH: Lactate dehydrogenase.

During the six-day admission course patient’s hemoglobin level improved and reached 9.2 mg/dL without any additional transfusion (Figure 3), and indirect bilirubin decreased to 1.3 mg/dL.

Active hemolysis seemed resolved, allowing for safe patient discharge. Prednisone was planned for a two-week taper with a decrease of 10 mg every two days until completion. The patient was to follow up with a primary care provider (PCP) and infectious disease (ID) specialist upon discharge, with a repeat CBC to be completed in one week.

The patient noted complete resolution of symptoms at one-week follow-up appointments with a PCP and ID specialist. One-week post-discharge, Hb improved to 11.3 mg/dL while still on treatment. Hemoglobin normalized by week three, 12.0 mg/dL, two weeks after treatment completion. The repeat tick-borne PCR panel on week three post-discharge was negative, including for babesia, with no detected parasite on smear evaluation. Serology noted a high positive titer (1:1024) for *Babesia microti*, with all other tick-borne titers being negative. The patient was back to his regular activities without limitation.

## Discussion

Babesiosis is primarily tick-borne, usually by Ixoides scapularis in the US, although transmission can also occur through blood transfusion, solid organ transplantation, and transplacental transmission [1-4,7]. Most cases are asymptomatic. Symptoms may present 1-8 weeks after transmission, typically within four weeks after a tick bite [5]. Fever, chills, malaise, and fatigue are the more common presenting symptoms and may be associated with arthralgia, myalgia, hepatosplenomegaly, and jaundice [2-6]. Laboratory findings of intraerythrocytic inclusion on blood smears, thrombocytopenia, mild hemolytic anemia, mild to moderately elevated liver enzymes, and renal injury are also commonly seen [2]. Severe cases may present with hemodynamic instability, multiorgan failure, altered sensorium, and death.

*Babesia microti* is the species most commonly responsible for babesiosis in the US. *Babesia spp.* only infects erythrocytes and can lead to hemolysis via two major pathways. The first pathway is cell membrane disruption and lysis while exiting the cell after the reproduction cycle [2,6]. The parasite lifecycle starts when an infected tick feeds on animals, most commonly rodents, and introduces sporozoites into the dead-end host (a human) during feeding. Sporozoites migrate to erythrocytes to facilitate asexual reproduction via budding. The budding process produces tetrad merozoites, which form the pathognomonic “Maltese cross” on giemsa staining. Merozoites rupture and exit the cell, leading to the lysis of RBCs and infecting other RBCs to continue the reproductive process. Symptoms develop as a result of this cycle [2,6,8]. The associated rate of anemia caused by this process is believed to be dependent on parasite load, RBC stability, and underlying host immunocompetence. Elderly and immunosuppressed patients have been observed to present with severe illnesses.

The second and rarer pathway is immune mediated, though there is no clear explanation for this process, with several theories proposed. One such theory is that infection causes a systemic inflammatory response leading to the deposition of immune complexes on RBCs, promoting premature destruction. Studies in mice have observed an early immune-mediated response against Babesia microti with the release of IL-2 and interferon-gamma, whereas anti-inflammatory cytokines IL-4 and IL-10 expression rise in the late phase, which mediates the host inflammatory response. Merozoites can also induce the release of inflammatory cytokines [9-11]. This heightened inflammatory response could lead to aberrant autoimmune activation. Alternative theories report possible cross-reactivity between RBC antibodies and antibodies developed against babesia antigen, leading to inappropriate deposition of immune complexes on RBCs and promoting their premature destruction [6,12].

Of the reported cases of babesia-related AIHA, the majority are due to wAIHA [12- 14]. Observed antibodies in wAIHA are almost always IgG; however, IgA and IgM have also been observed [1,15-18]. Once antibody deposition occurs, RBCs are mostly destroyed extravascularly via hepatic and splenic pathways.

In the splenic pathway, macrophages have Fc-gamma receptors that recognize and consume the IgG heavy chain and a portion of the red cell membrane, mainly responsible for red blood cell clearing. The outcome of each step is schistocyte and spherocyte formation (Figure 2). In the hepatic pathway, Kuppfer cells have receptors for complement C3 fragments and can phagocytose red cells with complement on their surface. The observed hepatosplenomegaly in our patient was likely the result of increased activity in RBC destruction as a result of this process.

The case presented is a rare presentation of babesiosis-associated wAIHA in an individual with a functioning spleen. Case reports reviewed involving babesia-associated wAIHA often involve asplenic and/or immunosuppressed individuals [12-14]. Splenic absence in these cases was noted to be associated with a more severe illness and the need for prolonged immunosuppressive therapies. Splenectomy is usually the last resort in the management of wAIHA, though it is the most effective, with ~70% remission. However, babesia-associated wAIHA pathophysiology seems to lead to more severe hemolysis, for which splenic presence seems to be more protective. It is likely that without splenic clearance of immune complexes deposited on RBCs, the accumulation of this complex in circulation leads to heightened complement cascade activation, leading to increased intravascular hemolysis and clearance via the hepatic pathway [12-14,19]. 

In babesia-associated wAIHA, a combination of standard therapy for babesiosis may be enough to mitigate the hemolysis; however, in some cases, adding standard wAIHA therapy may be indicated. The preferred treatment for B. microti infection is atovaquone plus azithromycin [5]. An alternative treatment of clindamycin plus quinine days may be used. Patients with either parasitemia 10% or greater, hemolytic anemia, or severe pulmonary, renal, or hepatic impairment are recommended for exchange plasmapheresis [5]. wAIHA-directed treatment may also be initiated to mitigate hemolysis in some cases. The first line of treatment for wAIHA is steroids, which may be augmented with immunosuppressive therapy with rituximab depending on severity and if there are contraindications to initiating rituximab [17].

Our patient noted daily symptom resolution with the initiation of treatment and clinical exam, particularly jaundice, which was resolved at the time of discharge. He was noted to have completely normalized blood work two weeks after treatment completion. It is likely that our patient’s quick recovery was due to functional spleen, early detection, and clinical suspicion. As with reported cases [12-14], treating both the wAIHA and babesia was necessary to prevent further hemolysis in the above case. 

## Conclusions

This case aims to raise clinical suspicion and consideration for babesiosis in elderly immunocompetent individuals who present with wAIHA regardless of spleen presence, particularly if they have been to endemic areas. A blood smear is an effective tool for early detection. Early detection and treatment can lead to dramatic clinical improvement for patients and decrease morbidity. In some cases, concurrent treatment for hemolysis and babesia may be necessary as well. As the number of reported babesiosis cases increases, there is a need to create more awareness of some atypical presentations that allow for early recognition and treatment.
